# miR-454-3p and miR-194-5p targeting cardiac sarcolemma ion exchange transcripts are potential noninvasive diagnostic biomarkers for childhood dilated cardiomyopathy in Egyptian patients

**DOI:** 10.1186/s43044-022-00300-x

**Published:** 2022-09-08

**Authors:** Alaaeldin G. Fayez, Nora N. Esmaiel, Sohair M. Salem, Engy A. Ashaat, Sonia A. El-Saiedi, Mona O. El Ruby

**Affiliations:** 1 Human Genetics and Genome Research Institute, National Research Centre, Dokki, Egypt; 2 Division of Pediatric Cardiology, Faculty of Medicine, Cairo University, Giza, Egypt

**Keywords:** Childhood dilated cardiomyopathy, Noninvasive diagnostic biomarkers, miRNAs, Cardiac sarcolemma, Gene ontology analysis

## Abstract

**Background:**

Childhood dilated cardiomyopathy (CDCM) is the most common cardiomyopathy in children and it is risk factor to heart failure and sudden death. Most of the different etiologic factors which have been postulated to DCM are idiopathic, and its pathogenesis remains uncertain. So it was worth investigating the potential DCM pathogenicity models to establish early noninvasive diagnosis parameters especially in CDCM patients. Beside that miRNAs in the circulatory blood are genetically considered the best option for noninvasive diagnosis; also, implementation of miRNAs as early diagnostic markers for children with DCM is urgent because those children have high risk to sudden heart death. We aimed to identify discriminator diagnostic circulatory miRNA expression levels in CDCM patients.

**Results:**

The expression levels of miR-454-3p and miR-194-5p were found significant upregulated (*p* value = 0.001 and 0.018; CI 95%, respectively), while miR-875-3p was found significant downregulated (*p* value = 0.040; CI 95%). A receiver operating characteristic (ROC) curve analysis showed significant AUC = 1.000 and 0.798 for miR-454-3p and miR-194-5p, respectively, and the optimal discriminated diagnostic cut-points were computed by index of union (IU) method. Enrichment analysis for the potential targeted mature mRNAs by miR-454-3p and miR-194-5p pointed that Ca, Na and K ions homeostasis in cardiac sarcolemma consider potential CDCM pathogenicity model.

**Conclusions:**

miR-454-3p and miR-194-5p are highly influencing noninvasive biomarkers for CDCM, and further circulatory miRNAs-implicated studies are highly recommended.

## Background

Younger adults and children are most frequent affected individuals by dilated cardiomyopathy (DCM), and patients with DCM are characterized by left ventricular or biventricular dilation and depressed systolic function without others abnormal loading conditions such as valve abnormalities, coronary artery disease (CAD) and hypertension [1, 2].

Idiopathic DCM is considered to be familial in 20 to 35% of cases, and international registry data indicate that DCM is the most common reason for heart failure and cardiac transplantation in pediatric [3, 4].

Remodeling of myocardial cells is continuous process by transduction of intercellular signals and activation of the transcription and transmission pathways, and DCM has been considered outcome of several pathological pathways [5].

MicroRNAs (miRNAs, miR) are a class of functional single-stranded noncoding RNA. Cytoplasmic miRNAs consider as gene expression regulators by modifying transcription or translational through targeting mainly the 3′ untransalted region (3′UTR) of mature mRNAs, and abnormal gene expression was addressed in various genetic disorders including cardiogenesis abnormalities, cardiac hypertrophy and electrical conduction impairment [6–12].

miRNAs studies in DCM revealed dysregulated miRNAs in cardiomyocyte, and also, bioinformatic analysis revealed that the downregulation of key pro-survival miRNAs promoted apoptotic signaling and heart decompensation [13, 14]. Few studies addressed plasma miRNAs profiles in CDCM, so identify unique signature in miRNA expression profiles in CDCM may lead to identify new early diagnostic noninvasive biomarkers as well as new therapeutic targets. More than 50 relevant genes have been identified in DCM, including in the genes encoding cytoskeletal, nucleoskeletal, mitochondrial, and calcium-handling proteins [15].

Circulating miRNAs would be best biomarker choices for early diagnosis of CDCM, because difficulties of tissue sampling in children. According to what given above and lack of noninvasive early diagnosis biomarkers to CDCM patients create a need for novel approaches to identify pathological mechanisms and support clinical decision-making, we aimed to characterize the profile of distinct plasma miRNAs expression in CDCM and analyze their target genes network using target gene ontology analysis. We assessed the difference of the studied miRNAs between CDCM patients and the corresponding controls; also, sensitivity, specificity and cut-point values of miRNAs expression were computed.

Reasons of targeting of the Egyptian CDCM patients in the current research were (i) what was recently reported that genetic makeup of cardiomyopathy differs from ethnic to other [14]. (ii) It was found that miRNAs have differentiated profile in diseases between African and European descendants, which could be responsible for differences among those populations in susceptibility to diseases, drug sensitiveness, and biomarker diagnostics [16, 17].

## Methods

*Patients*: A total of 37 CDCM patients and 37 non-cardiac anomalies healthy controls whose age ranged from 9 months to 18 years old were recruited from the Excellence Center of Medical Research, Clinical Genetics Department, CHD Clinic and pediatric department, Cairo university.

*Clinical investigations*: Full clinical examination of cases and control groups were done for each case with special emphasis on heart and cardiovascular system. Family history of any similarly affected person in the family and consanguinity were reported. Others possible DCM causes were excluded such as coronary artery disease (CAD) which investigated with coronary angiography and hypertensive heart disease.

*Blood sampling:* For diagnosed DCM cases and healthy individuals, blood samples had been taken into a sterile vacutainer tube with anti-coagulant factor (pot. EDTA) after the agreement signature on the consent form the children’s parents or guardians.

*Blood processing and storage*: All of the blood samples were centrifuged at 3000 rpm for 15 min to collect the plasma. Then, the plasma was aliquoted in the RNase-free microfuge tubes and store at − 80 centigrade.

*RNA extraction and RT-PCR:* Total plasma miRNAs were isolated using Direct-zol™ RNA MiniPrep (Zymo Research) according to manufacture’s protocol. Each RNA sample was quantified with a spectrophotometer (NanoDrop 1000, Thermo Scientific, Wilmington, Delaware). 20 ng of the isolated miRNAs were converted to complementary DNA (cDNA) by miScript II RT Kit (Qiagen). qRT-PCR reactions were conducted in 96-well plates with 120 ng/ul of the cDNA and miScript SYBR Green PCR Kit (Qiagen) using ROCH 480II lightcycler instrument. Quantitative real-time polymerase chain reactions will be performed in duplicate for all samples. miRNAs PCR primers will be provided upon request.

*qRT-PCR data analysis:* The data were analyzed using the relative fold change (FC) by ∆∆*Ct* equation and transformed log2FC values. Relative gene expression level was calculated by 2^−(∆∆*Ct*)^ equation, and miRNA expression levels were normalized to a nonendogenous synthetic miRNA miR-16-2.

*Target genes analysis:* To better understand the biological function of the significant dysregulated miRNAs, their putative target genes were predicted under confidence interval 95% using TargetScan [18], miRDB [19], MiRTarBase 9.0 [20] and miRWalk [21] databases; to gain deep insight into the biological functions of the differential miRNAs and target genes, the pathways of target genes will be analyzed by KEGG [22] database and protein–protein interaction (PPI) enrichment *p* value was computed by STRING tool [23].

*Statistical analysis*: Values are expressed as mean ±SD or median according to skewness and kurtosis values, and outliers’ values were excluded. Student's two sided t test was   used for   mean miRNAs expression comparison. Receiver operating characteristic (ROC) curves were analyzed to assess specificity and sensitivity of each single plasma miRNA. The optimal diagnostic cut-point of the significant miRNA was assessed by index of union (IU). *p* < 0.05 value considered significant. The statistical analysis was done by PASW statistics [24] (SPSS Inc. Released 2009. PASW Statistics for Windows, Version 18.0. Chicago).

## Results

*Clinical characteristics of patients:* A total of 37 CDCM patients were enrolled in this study as diagnosed by the Canadian Cardiovascular Society guidelines, beside 37 age-matched non-cardiac anomalies healthy as controls. The clinical characteristics of patients are shown in Table 1.

**Table 1:** Patients and control characterization

Group	*n*	Age (years)* Mean ± SD	Gender (%)	Consanguinity (%)
CDCM	37	9.5 ± 2.5	Male; 55 Female; 45	+ve (76%) −ve (24%)
Control	37	8 ± 3	Male: 47 Female; 53	NA

*No statistically significant difference was found between CDCM and control groups for ages, *p* value = 0.089

**Fig. 1:**
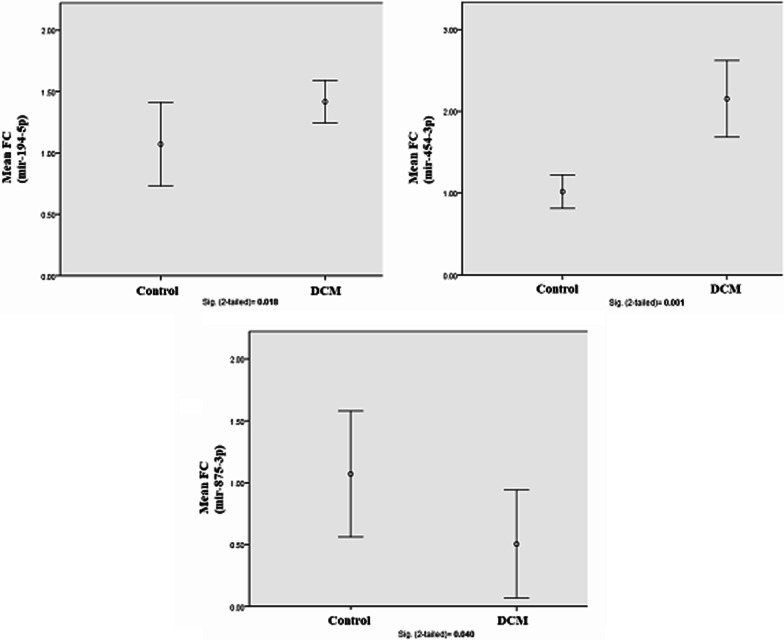
Error bars for distribution of fold change (FC), quantified by ∆Ct, of plasma miR-194-5p, miR-454-3p and miR-875-3p between DCM cases and controls. *p* value was calculated by independent t test; significant *p* value ≤ 0.05 at CI = 95% was considered

*Both miR-454-3p and miR-194-5p were significant upregulated and miR-875-3p was significant downregulated in CDCM patients:* To identify miRNAs that are differentially expressed in CDCM and control, we performed real-time PCR with duplicate expression reactions to five selected miRNAs that are miR-518-3p, miR-618, miR-875-3p, miR-454-3p, miR-194-5p from CDCM patients and controls. By applying a criterion of fold change (FC) to assess the differential miRNAs expression ratio between CDCM and control groups, it was found that fold expression change of miR-454-3p and miR-194-5p was significantly higher in CDCM patients than the control subjects. On contrast, miR-875-3p was significantly lower in CDCM patients than the control subjects as shown in Fig. 1. There was a significant correlation between CDCM occurrence and each of miR-454-3p, miR-194-5p and miR-875-3p; therefore, these miRNAs could be useful as potential diagnostic parameters to CDCM patients. Both miR-518-3p and miR-618 showed insignificant fold expression change difference between CDCM and control groups.

**Fig. 2:**
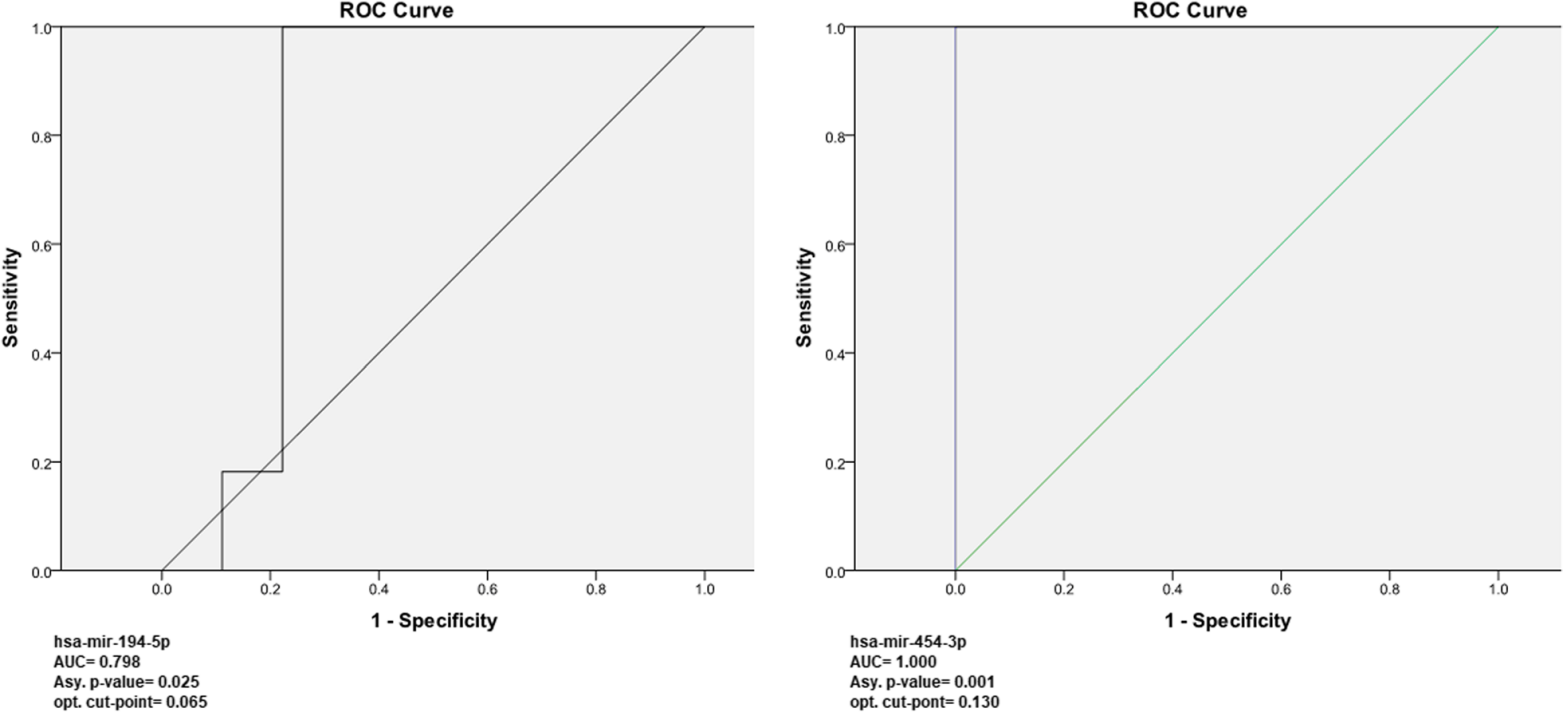
Receiver operating characteristic (ROC) curve of circulating mir-194-5p and 454-3p for the diagnosis DCM

*Diagnostic value assessment of miRNAs for CDCM; both miR-194-5p and miR-454-3p have significant discriminative cut-point values*: To discriminate between the CDCM patients and the healthy controls, the receiver operating characteristic (ROC) curves were plotted for every single miRNA log2FC values. The areas under the curves (AUCs) were 0.798 and 1.000 for mir-194-5p and 454-3p, respectively. The measured optimal cut-points for them were calculated using index of union (IU) method, where 0.065 and 0.130 were the optimal discriminator boarder value for mir-194-5p and 454-3p respectively discovering the CDCM patients. These results are suggesting that these circulating miRNAs may be useful for CDCM detection and as noninvasive early diagnosis predictors as shown in Fig. 2.

*Kyoto encyclopedia of genes and genomes (KEGG) pathways analyses:* To predicted putative target genes of the differentially expressed miRNAs, analysis according to miRWalk tool, including TargetScan, mirDB and MirTarBase tools, was used to classify and annotate both miR-194-5p and miR-454-3p according to two KEGG pathways that are (i) cardiac muscle contraction (hsa04260) and (ii) DCM (hsa05414) over three genome regions, 3′UTR, 5′UTR and CDS as shown in Table 2. The predicted target genes are shown in Tables 3 and 4. It is worth mentioning that 3'UTR region of the targeted TGFB2 gene was found as common targeted gene between miR-194-5p and miR-454-3p with differentially binding energy − 23.6 and − 18.3, respectively.

**Table 2:** miRNA-target genes analysis according to the relevant KEEG pathways (miRWalk analysis)

	Targeted gene regions		
	3′UTR	5′UTR	CDS
*(I) Cardiac muscle contraction (hsa04260)*			
miR-454-3p	+	−	+
miR-194-5p	+	−	+
*(II) Dilated cardiomyopathy DCM (hsa05414)*			
miR-454-3p	+	−	+
miR-194-5p	+	−	+

Under score ≥ 0.95

**Table 3:** Predicted targeted genes for miR-454-3p (based on MANE transcript)

Gene	MANE transcript	Binding probability	Binding energy	AU rich region fraction	Positions	KEEG pathways
CACNB2	NM_201590	0.953846154	− 20	0.75	3′UTR	Cardiac muscle contraction (hsa04260)
	NM_201596	0.953846154	− 20	0.75	3′UTR	
ATP1A4		1	− 19.6	0.485	CDS	
CACNB2	NM_201590	0.953846154	− 20	0.75	3′UTR	DCM (hsa05414)
	NM_201596	0.953846154	− 20	0.75	3′UTR	
TGFB2	NM_003238	1	− 18.3	0.529	3′UTR	
ITGB6	NM_000888	1	− 19.8	0.5	CDS	
ITGA2B	NM_000419	1	− 19.1	0.471	CDS	
*Common target gene in the used KEEG pathways*						
CACNB2						

It means matched annotated transcripts from refseq (NCBI) and gencode (Ensemble;EMBL-EBI)

**Table 4:** Predicted targeted genes for has-mir-194-5p (based on MANE transcript)

Gene	MANE Transcript	Binding probability	Binding energy	AU rich region fraction	Positions	KEEG pathways
CACNA2D1	NM_000722	1	− 20.5	0.588	3′UTR	Cardiac muscle contraction (hsa04260)
CACNA1C	NM_000719	0.991452991	− 22.1	0.456	3′UTR	
	NM_001167623	0.991452991	− 22.1	0.456	3′UTR	
ATP1B3	NM_001679	1	− 17.6	0.515	CDS	
ATP1A1	NM_000701	1	− 21.9	0.5	CDS	
RYR2	NM_001035	1	− 20.5	0.5	CDS	
CACNA2D1	NM_000722	1	− 20.5	0.588	3′UTR	DCM (hsa05414)
CACNA1C	NM_000719	0.991452991	− 22.1	0.456	3′UTR	
	NM_001167623	0.991452991	− 22.1	0.456	3′UTR	
TGFB2	NM_003238	1	− 23.6	0.529	3′UTR	
DAG1	NM_004393	1	− 19.3	0.441	3′UTR	
TTN	NM_001267550	1	− 22.8	0.515	CDS	
ITGA2	NM_002203	1	− 18.3	0.691	CDS	
RYR2	NM_001035	1	− 20.5	0.5	CDS	
*Common target genes in the used KEEG pathways*						
CACNA2D1, CACNA1C, RYR2						

It means matched annotated transcripts from refseq (NCBI) and gencode (Ensemble;EMBL-EBI)

**Fig. 3:**
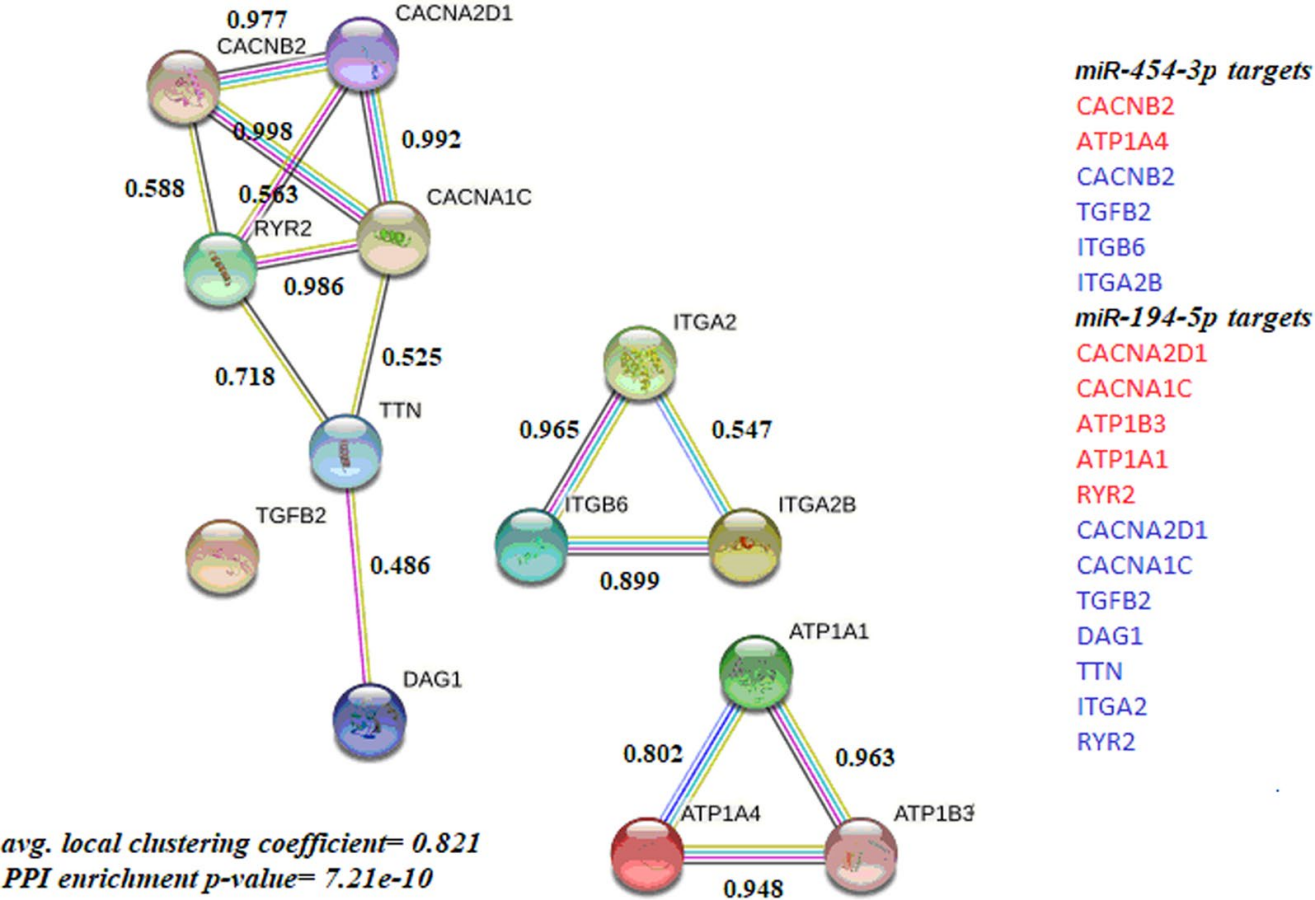
PPI enrichment network for the whole target genes including interaction score on edges lines. On the right column, proteins by red highlighted refer to cardiac muscle contraction pathway relevant targets and by blue highlighted refer to DCM pathway relevant targets. PPI enrichment *p* value of miR-454-3p = 0.181, PPI enrichment *p* value of miR-194-5p = 6.09e-06

To detect the relationship between overall targeted genes by miR-194-5p and miR-454-3p, protein–protein interaction (PPI) enrichment was computed by STRING tool; there are significant PPI enrichment network found as shown in Fig. 3.

**Fig. 4:**
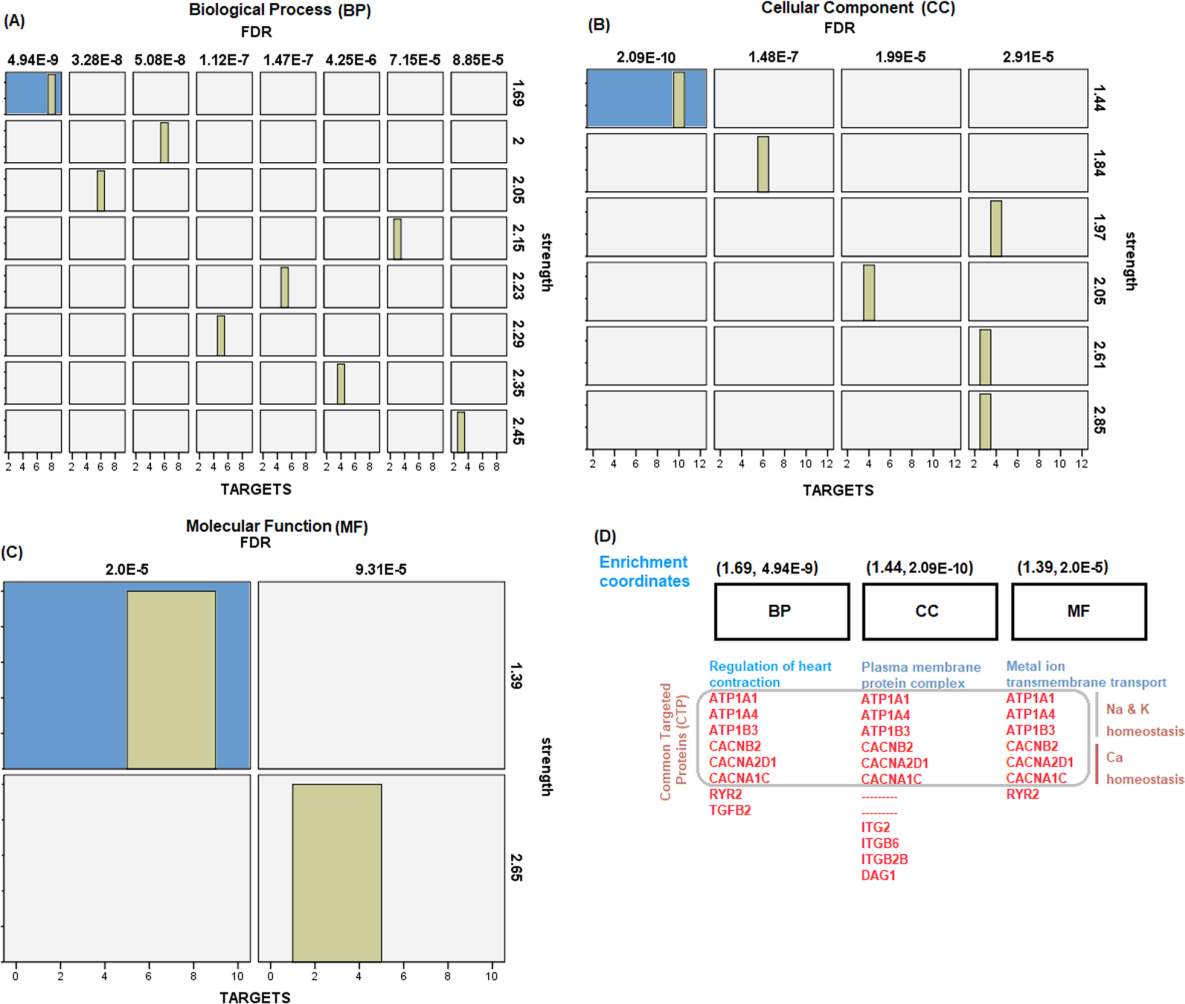
Differential PPI enrichment network according to the biological process (**A**), cellular components **B** and molecular function (**D**). The blue square highlighted refer to the best coordinate containing lowest FDR, highest number of protein as targets and followed by strength. **C** Represents intersected enrichment coordinates showing highly potential role of calcium (Ca), sodium (Na) and potassium (K) ions homeostasis cross the cardiac sarcolemma in CDCM pathogenicity

Differential PPI enrichment network was done by intersection classification of the targeted proteins across three classifiers that are (i) strength (log10 [observed/expected]), (ii) false detective rate (FDR) and (iii) number of the targeted proteins as shown in Table 5 and Fig. 4a, b, c. The intersection classification showed that miR-194-5p and miR-454-3p might cause CDCM via impairment of calcium, potassium and sodium homeostasis through plasma membrane of a cardiac muscle fiber cell (sarcolemma) as shown in Fig. 4d.

**Table 5:** GO analysis for whole targeted genes (*n* = 13) by miR-194-5p and miR-454-3p using STRING tool

GO	FDR*
*Cellular components (CC)*	
Sarcolemma	2.11e-0.6
T-tubule	0.00052
Ca channel complex	0.00058
Molecular function (MF)	
Alpha actinin binding	0.0014
Sod–pot exchange	0.0064
Biological process (BP)	
Cell communication by electric coupling involved in cardiac contraction	4.19e-05
Ca ion transport	0.00069
*Relevant pathway prioritization*	
KEEG pathway	
DCM	1.4e-10
HCM	1.4e-10
Arrhythmogenic RV cardiomyopathy	8.98e-09
Cardiac muscle contraction	1.29e-08
Reactome pathway	
Muscle contraction	9.56e-05
Cardiac conduction	0.0011
Wiki pathway	
Arrhythmogenic RV cardiomyopathy	9.13e-08
Ca regulation in cardiac cell	0.0214

*FDR; False Detection Rate

## Discussion

In the present study, 5 circulating miRNA biomarkers were selected from the CDCM-relevant literature to asses them as diagnostic biomarkers which can use as noninvasive early diagnosis. Notably that studies that conducted on plasma or serum miRNAs were rare. Plasma miRNAs were extracted to determine its relative expression levels by qRT-PCR in 37 CDCM patients against age-matched 37 DCM- free controls. The main findings of this study can be summarized as follows: (i) high positive consanguinity was observed, (ii) human miR-194-5p and miR-454-3p upregulation is potential noninvasive biomarkers for CDCM patients; (iii) ion exchange and cardiac electric conduction through cardiac sarcolemma are potential affected cellular components resulted from miR-194-5p and miR-454-3p overexpression and lead to CDCM pathogenicity; (iv) according to PPI enrichment network and GO analysis, there are 13 targeted genes may be affected by mir-194-5p and mir-454-3p overexpression, and they commonly were ATP1A1, ATP1A4, ATP1B3, CACNA2D1, CACNA1C and CACNB2 genes.

Many different etiologic factors were described to cause myocardial damage in DCM [25]. According to cardiomyopathies-relevant Egyptian studies, positive consanguinity could be risk factors in the cardiomyopathies etiology, where Darwish et al. [26] reported a first study to uncovering the genetic background of idiopathic primary hypertrophic cardiomyopathy in 24 Egyptian patients with 62.5% positive consanguinity; they pointed the high burden of consanguinity in Egyptian pediatric hypertrophic cardiomyopathy might associate with particular genetic background. Mehaney et al. [27] pointed that 53% of the studies dilated cardiomyopathy patients showed positive consanguinity, and the authors suggested that high burden of consanguinity might lead to novel genes or variants underlie pediatric cardiomyopathy in Egyptian DCM patients.

miRNAs consider as key players in idiopathic dilated cardiomyopathy, where Bioinformatic analysis revealed that the downregulation of key pro-survival miRNAs promoted apoptotic signaling and heart decompensation [28, 29].

In 2008, Sucharov et al. [30] analyzed the miRNA expression profile by microarray in tissue samples from idiopathic DCM and ischemic DCM patients. The authors identified a profile of miRNAs deregulated in both phenotypes that were miR-100 and miR-195 overexpression and miR-92 and miR-133b downexpression. Zhou et al. [31] pointed that expression levels of miR-208b was upregulated in myocardium samples. Shen et al. [32] mentioned that miR-146a could be a useful protective agent against sunitinib-induced cardiac dysfunction.

In plasma miRNAs profiling in idiopathic DCM, Coskun et al. [25] pointed that miR-618, miR-875-3p, and miR-194 were found decreased, while expression levels of miR-518f and miR-454 were found increased in DCM patients to consider that these miRNAs as potential diagnostic biomarkers, and these results are agreed with what were pointed in the current research except miR-194 expression which may due to different sample size or clinical traits spectrum. However, Steer and Subramanian [33] pointed that miR-875-3p expression level was similar in adult heart failure patients to those in healthy controls. This inconsistency regarding miR-875-3p expression level may due to different sample size, clinical traits spectrum, and different etiology background of which have been demonstrated to cause DCM. As far as myopallidin gene was reported to harbor DCM-relevant variants, and this gene is targeted by miR-875-3p [34], and this may be support our resulted concerning miR-875-3p downregulation in children with DCM.

From a given above, implementation of miRNAs in early diagnosis is urgent for children with DCM, where there are high risk factor to sudden heart death and also that the circulating miRNAs in the plasma is considered the first option for diagnosis because it is noninvasive tool [35]. According to our knowledge, study of plasma miRNAs in Egyptian CDCM was not studied yet, so the current study might be first one in Egyptian CDCM patients.

qRT-PCR assay, in the current study, showed that miRNA alterations were well correlated with the CDCM patients, where both miR-194-5p and miR-454-3p have positive relationships with CDCM, and on contrast, miR-875-3p has negative relationship. Also, PPI enrichment network and GO analysis showed that there are 13 targeted genes could be affected by miR-194-5p and miR-454-3p, out of them 6 target genes, ATP1A1, ATP1A4, ATP1B3, CACNA2D1, CACNA1C and CACNB2, shared in CDCM pathogenicity as consequences of miR-194-5p and miR-454-3p overexpression. Consequently, overexpression pattern of miR-194-5p and miR-454-3p might lead to downexpression and translational repression of ATP1A1, ATP1A4, ATP1B3, CACNA2D1, CACNA1C and CACNB2. Therefore, what are the potential biological role of deregulated ATP1A1, ATP1A4, ATP1B3, CACNA2D1, CACNA1C and CACNB2 in CDCM pathogenicity?

From our GO analysis, CACNA1C and CACNB2 involved in voltage-depended calcium ion change. Molina-Navarro et al. [36] found that both CACNB2 and CACNA1C as cardiac ion channel genes were significantly downregulated in LV biopsies of DCM patients against normal heart tissue.

Our miRNAs-targets gene analysis concluded that coding sequence of MANE transcript (NM_000701) for ATP1A1 gene and coding sequence of MANE transcript (NM_001035) for RYR2 gene are targeted by miR-194-5p with high binding probability and binding energy − 21.9 and − 20.9, respectively. Guo et al. [37] found that mRNA levels of ATP1A1 were significantly higher (*p* value = *p* < 0.05) in the pediatric and adult DCM group than the control group. Stanczyk et al. [38] revealed experimentally Ca^2+^ homeostasis impairment consider pathogenic mechanism contributing to the development of DCM, HCM and/or arrhythmogenicity.

isolated cardiomyocytes from neonatal rats were isolated by Li et al. [39] to explore role of dysregulated miRNAs in the initiation and progression of myocardial ischemia–reperfusion (MI/R) in a calcium-dependent manner; they found that downregulation of miR-202-5p led to Trpv2 upregulation inhibiting Ca^2+^ overload in cardiomyocytes. Belevych et al. (2011), Xu et al. (2012), Curcio et al. (2013), Liao et al. (2016) revealed experimentally that miRNA-1 drives arrhythmogenesis by altering ion channels and proteins associated with the heart’s electrical activity in cardiac diseases [40–43]. Also, Wiedmann et al. [44] aimed to study differential expression of selected miRNA in atrial tissue samples obtained from patients with sinus rhythm, paroxysmal AF, or permanent/chronic AF, and they found that miR‐25, miR‐21, miR‐34a, miR‐23a, miR‐124, miR‐1, and miR‐29b upregulation as well as miR‐9 and miR‐485 downregulation were associated with TASK‐1 potassium channel in patients with atrial cardiomyopathy. The above results conclude the disturbance of cardiac ion channel proteins resulting from dysregulated miRNAs may be one of cardiac diseases pathogenesis.

According to the current study, Ca, Na and K homeostasis impairment might be potential pathogenicity effect to cause CDCM. Feng (1992) showed that erythrocyte membrane Na-pump and Ca-pump were remarkably lower in DCM than those in the controls [45], Plank et al. [46] found 3.6-fold reduction of sarco(endo)plasmic reticulum Ca2 + -ATPase (SERCA) in cardiomyocytes, El-Battrawy et al. [47] found significant reduction of systolic and diastolic intracellular Ca^2+^ concentrations was detected in DCM cardiomyocytes, but potassium concentration was similar in DCM and control cardiomyocytes, and Ednie et al. [48] pointed that altered glycosylation contributes to DCM through changes in Na_*v*_ and K_*v*_ activity that impact conduction Ca^2+^ handling and contraction.

## Conclusions

Finally, we concluded that both miR-194-5p and miR-454-3p were significantly upregulated in plasma of the studied CDCM targeting 5 potential MANE transcripts involved in Ca, Na and K homeostasis in cardiac sarcolemma according to the current research limits.

## Data Availability

The datasets used and/or analyzed during the current study are available from the corresponding author on reasonable request.

## Abbreviations

CDCM: Childhood dilated cardiomyopathy
DCM: Dilated cardiomyopathy
miRNA: Micro-Ribonucleic acid
ROC: Receiver operating characteristic
AUC: Area under curve
IU: Index of union
CAD: Coronary artery disease
3'UTR: 3′ Untransalted region
5'UTR: 5′ Untransalted region
CDS: CoDon sequence
CHD: Congenital heart defect
KEGG: Kyoto encyclopedia of genes and genomes
PPI: Protein–protein interaction
FDR: False detective rate
MANE: Matched annotated transcripts between NCBI and ensemble
GO: Gene ontology
